# Human *in vitro* Model Reveals the Effects of Collagen Cross-linking on Keratoconus Pathogenesis

**DOI:** 10.1038/s41598-017-12598-8

**Published:** 2017-10-02

**Authors:** Rabab Sharif, Jesper Hjortdal, Henrik Sejersen, Garett Frank, Dimitrios Karamichos

**Affiliations:** 1 Department of Cell Biology, University of Oklahoma Health science Center, Oklahoma City, Oklahoma USA; 2 Department of Ophthalmology, Aarhus University Hospital, Aarhus C, Denmark; 3 Department of Ophthalmology/Dean McGee Eye Institute, University of Oklahoma Health Science Center, Oklahoma City, Oklahoma USA

## Abstract

Keratoconus (KC) is a corneal thinning disorder that leads to severe vision impairment As opposed to corneal transplantation; corneal collagen crosslinking (CXL) is a relatively non-invasive procedure that leads to an increase in corneal stiffness. In order to evaluate the effect of CXL on human corneal stromal cells *in vitro*, we developed a 3-D *in vitro* CXL model, using primary Human corneal fibroblasts (HCFs) from healthy patients and Human Keratoconus fibroblasts (HKCs) from KC patients. Cells were plated on transwell polycarbonate membranes and stimulated by a stable vitamin C. CXL was performed using a mixed riboflavin 0.1% PBS solution followed by UVA irradiation. Our data revealed no significant apoptosis in either HCFs or HKCs following CXL. However, corneal fibrosis markers, Collagen III and α-smooth muscle actin, were significantly downregulated in CXL HKCs. Furthermore, a significant downregulation was seen in SMAD3, SMAD7, and phosphorylated SMADs -2 and -3 expression in CXL HKCs, contrary to a significant upregulation in both SMAD2 and Lysyl oxidase expression, compared to HCFs. Our novel 3-D *in vitro* model can be utilized to determine the cellular and molecular effects on the human corneal stroma post CXL, and promises to establish optimized treatment modalities in patients with KC.

## Introduction

**Figure 1:**
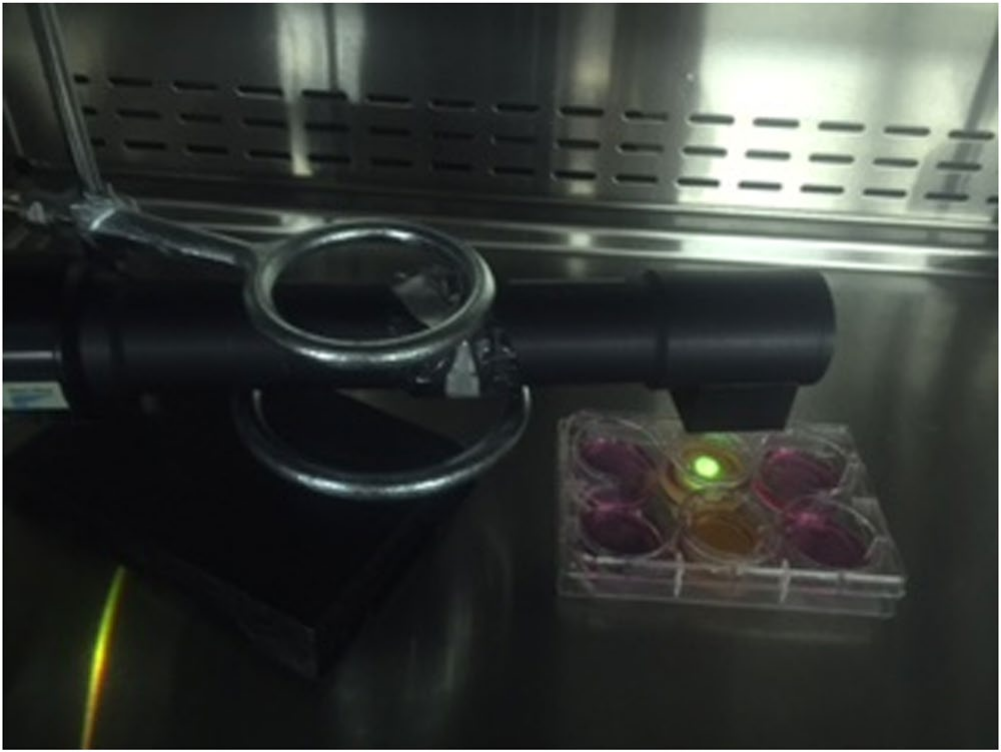
UVX-1000 illumination system/3-D *in vitro* model.

Keratoconus (KC) is a bilateral progressive disorder of the eye, characterized by thinning, scarring, and protrusion of the central cornea^1^. These defects in corneal extracellular matrix (ECM) assembly lead to myopia, and irregular astigmatism, which eventually advance to severe visual impairment. The prevalence in the general population is 50-200 per 100000^2^, and is typically diagnosed in the patient’s adolescent years^3^. KC is considered one of the foremost clinical indications for corneal transplants worldwide. Although a large number of clinical studies have been conducted the exact underlying KC pathobiology remains unclear. Considering there is currently no acceptable animal model for KC, we utilized our established 3-D *in vitro* model shown in (Fig. 1) to study cellular and molecular responses following radiation-induced corneal collagen crosslinking (CXL). Our model has been established for KC studies since 2012^4–12^ and provides a novel platform to study the cellular and molecular dynamics following CXL treatment. As well as guide us towards dissecting the KC pathobiology root^4^. The favored right angled collagen fibril orientation of the normal human cornea is severely impaired in KC^13,14^, mechanically revealing a substantial reduction in corneal stiffness, contributing to a biomechanical unstable corneal environment^15^. Furthermore, KC is characterized by a disruption in the balance between collagen production and proteolytic breakdown^16^. Both the concentration and the activity of the crosslinking enzyme (LOX) have been shown to be significantly reduced in KC corneas^17,18^.

In the last decade CXL has become a fundamental treatment for progressive ectasias including KC, and has recently been approved by the US Food and Drug Administration (FDA). Briefly, CXL exploits the combined properties of ultraviolet A (UVA, 370 nm) and riboflavin,^19–21^ which works as a photosensitizer for the induction of crosslinks between collagen fibrils. At the same time acts as a shield from the penetration of UVA in the underlying tissues^22^. UV irradiation excites the fluorescent molecule to a triplet state, with consequent generation of a singlet oxygen and superoxide radical^19^. These radical products are then able to strengthen the corneal stromal collagen bonds and increase resistance to enzymatic degradation forming covalent bonds between the amino acids of the adjacent collagen fibers^23^.

CXL was introduced in 2003 by Wollensak *et al*.,^24^ and has become a widely known, low invasive intervention with high success and low complication rates. The Dresden protocol, is a common cross-linking standard technique^25^, which involves the removal of corneal epithelium (epi-off) followed by riboflavin and UV-A irradiation at 3 mW/cm^2^. CXL without epithelial debridement (epi-on) technique has also been attempted and aims to reduce the risk of infection and post-operative pain, associated with epi-off^26^. Both techniques have been proven to be effective however, studies regarding safety and long term adverse effects are inconclusive^27^. In this study, our *in vitro* system lacks the integrity of the epithelial layer in order to simulate “standard” clinical settings.

Long-term stabilization and improvement after CXL have been reported in many prospective studies; however, failure and progression of keratectasia after CXL have also been reported^28^. The best candidate for CXL therapy are patients with a progressive KC^29,30^, but who also satisfy the following criteria; patients are recommended to be between the ages 16 and 40 years^31^, with a minimum corneal thickness of 400 microns, maximal keratometry <60 D based on Pentacam readings, and have no other known corneal disease^19^. CXL is not the ultimate treatment for KC, since little information is known in regards to the safety and long term efficacy of CXL in these patients and is not suitable for every keratoconus patient^19,32,33^. Nevertheless, treatment parameters should be tailored towards patients with a clear KC progression status. It is essential to elucidate as completely as possible the precise molecular effects of CXL, not only on the exposed resident cells but also on the ECM components.

The signaling pathway of transforming growth factor-β (TGF-β) is an intricate signal transduction cascade, that plays an important role in the alteration and production of the ECM in KC^34^. TGF-β has been identified as an important growth factor involved in the development of corneal fibrosis and scarring, as it activates corneal keratocytes, and promotes fibrosis represented by an increase in collagen type III and α-SMA expression^35^. Critical elements in the TGF-β signal transduction are the SMAD proteins, known to be modulated in KC^10^. The signal cascade is rather complex and extensively investigated. Briefly, upon TGF-β binding to its receptor, serine-threonine kinase receptors are activated and then bind to the receptor-activated SMADs (R-SMADs), SMAD2, and SMAD3^36^. Consequently, R-SMADs are phosphorylated and form a complex with the common SMAD4, translocating into the nucleus where they regulate transcription of TGF-β target genes^36,37^. The inhibitory SMAD7 is known to bind to TGF-β receptor competitively and interferes with the activation of SMAD2 and SMAD3 leading to an inhibition in the TGF-β signal transduction^10^. Inhibiting TGF-β adverse activity, through SMAD7 could mitigate an excessive wound healing reaction from ECM deposition, and myofibroblasts formation^36^. Thus, TGF-β/SMAD modulation in the cornea and KC could have therapeutic potentials for improvement of excessive corneal fibrosis and scarring.

Thus, in this study we aim to determine the effects of pre/post CXL on both HCFs and HKCs using our established 3-D *in vitro* model. The long term implications of our study are important for KC patients since it could establish optimized treatment modalities in these patients.

## Results

### Cell Viability

**Figure 2:**
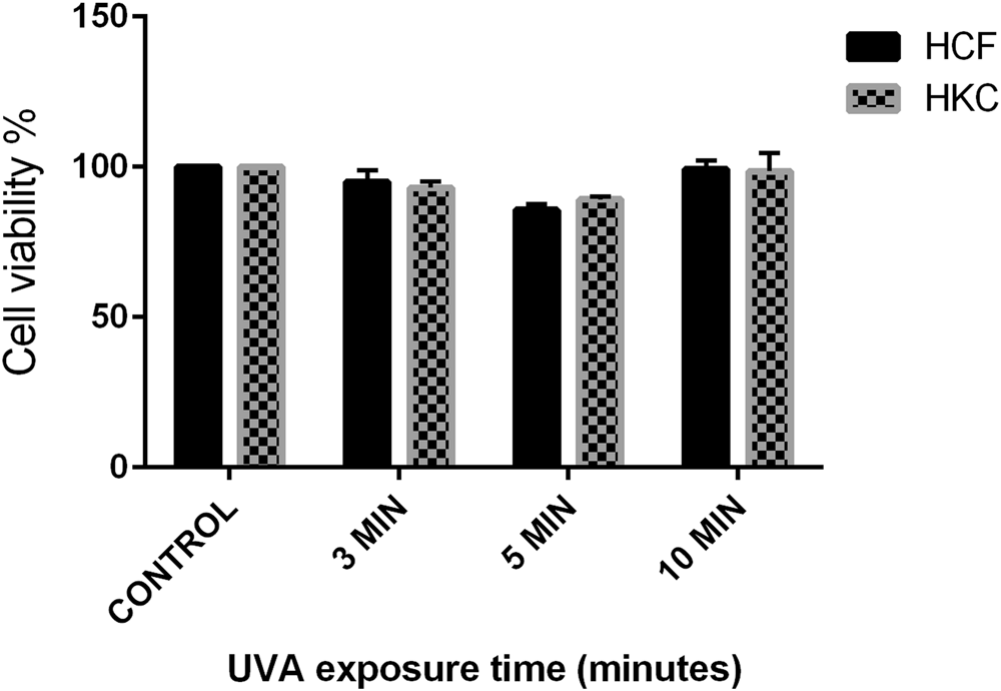
Live/Dead assay: shows the effect of CXL on corneal cell viability, HCF, and HKC were exposed to UVA irradiation + riboflavin for three different time periods (3 min, 5 min, and 10 minutes).

To determine the effects of CXL on HCFs and HKCs, the live/dead® Viability Assay kit (Molecular Probes, Eugene, OR) was used. All cultures were exposed to an excitation wavelength of 560 nm, and the emission at 616 nm was recorded using a 96-well microplate reader. We aimed to determine the percentage of live/dead KC cells compared to healthy cells following CXL for 3, 5, and 10 minutes (Fig. 2). This assay revealed slight increase in the percentage of dead HKC.X versus HCF.X, yet, not significant enough to cause dosage toxicity (*P > 0*.*35*). CXL showed no significant effect on HCFs viability.

### Cell Proliferation

**Figure 3:**
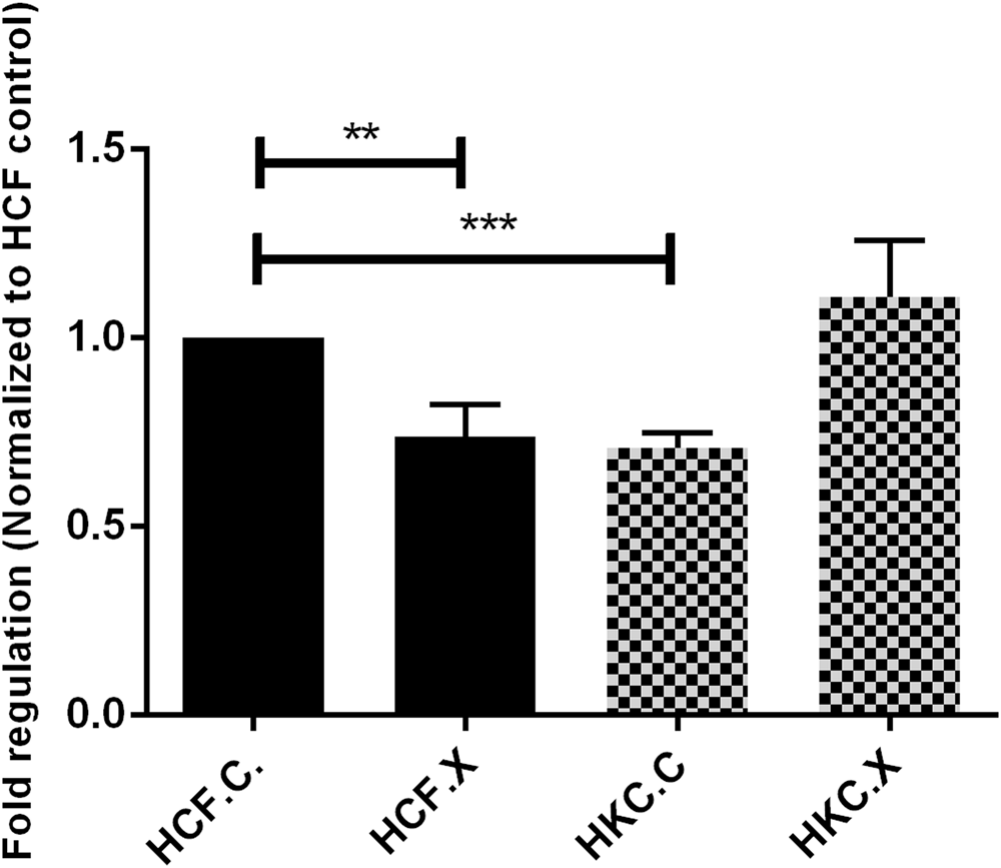
MTT assay quantification: HCF, HKC controls, and HCF and HKC CXL. Data was normalized to HCF controls and a fold regulation is plotted. One way ANOVA for a total n = 4 data sets. *(P = 0.0015), **(P = 0.0086), ***(P = 0.0008).

In order to measure cell proliferation rate and conversely, when metabolic events lead to apoptosis or necrosis, the reduction in cell viability^38^, the Vybrant® MTT proliferation assay kit was used in this study. Our data was expressed as fold regulation as previously determined in various studies^39–41^. Proliferation rate of HKCs, displayed in (Fig. 3), shows that 24 hours after CXL proliferation of HKC.X was increased significantly (*P = 0*.*0015*), compared to HKC.C. Repopulation by proliferating cells was observed 24 hours post CXL; these modifications are the morphological correlate of the process leading to an increase in biomechanical stability.

### Corneal Hydration post CXL

**Figure 4:**
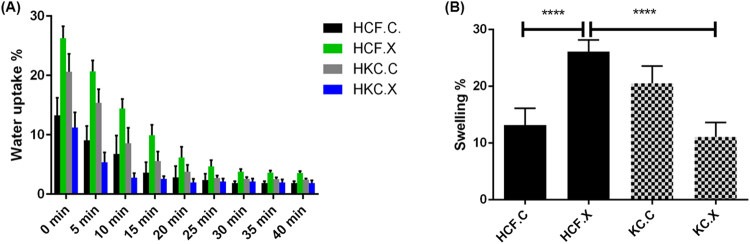
Cell hydration profile post CXL. Figure (**A**) represents that water uptake percentage quantified over a period of 40 minutes. Figure (**B**) shows the swelling ratio in both cell types after CXL treatment.

We evaluated how hydration status and CXL simultaneously affect corneal stiffness, we determined that the decreased hydration of cross-linked constructs, contribute to an increase in ECM stiffness. Our data reveals a higher hydration % in the HKC.C compared to HKC.X (Fig. 4). On the other hand, water loss transition in the HKC.X was accelerated compared to HKC.C. However, in comparison to healthy corneal cells, HCF.X versus HCF.C showed higher matrix hydration %, but the water loss transition level for both conditions plateaued around 20 minutes post CXL (Fig. 4A). In previous studies^16,42^, hydration status of corneal samples was determined by calculating percentage water content in each sample. The water uptake percentage of the HKC.X tend to be less than the HCK.C, and the water loss transition in these cells is faster, due to the reinforced biomechanical stability obtained through CXL. Furthermore, HCF.X tend to take up more water reflected in a higher swelling ratio when compared to HKC.X *(P < 0*.*0001)*, as shown in (Fig. 4B). This could be explained by the fact that HCFs are expressing a weaker and less stable ECM when exposed to CXL, a state that somewhat resembles the KC cornea environment.

### Cell Migration post CXL

**Figure 5:**
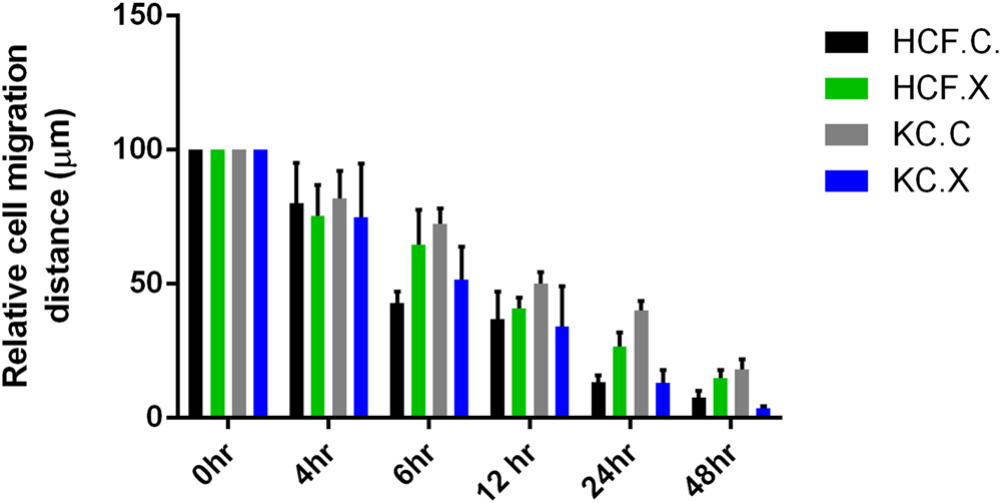
Cell migration: HCFs, and HKCs were scratched and the relative cell migration distance was quantified at 0 hr, 4 hr, 24 hr and 48 hr time points.

We analyzed and quantified cell migratory pattern for both cell types following CXL, compared to their respective controls. Using an *in vitro* scratch assay model^43^, we calculated the distance that cells had traveled into the wound area using the cell monolayer’s leading edge. We observed a significant 2 fold increase in cellular migration (*P ≤ 0*.*0001*) in HKC.X when compared to HKC.C (Fig. 5). This suggests that HKC.X have the ability to potentially perform normal wound closure. However, no significant difference was observed between HCF.C and HCF.X.

### Effect of CXL on specific ECM markers expression

#### α-SMA expression pre/post CXL

**Figure 6:**
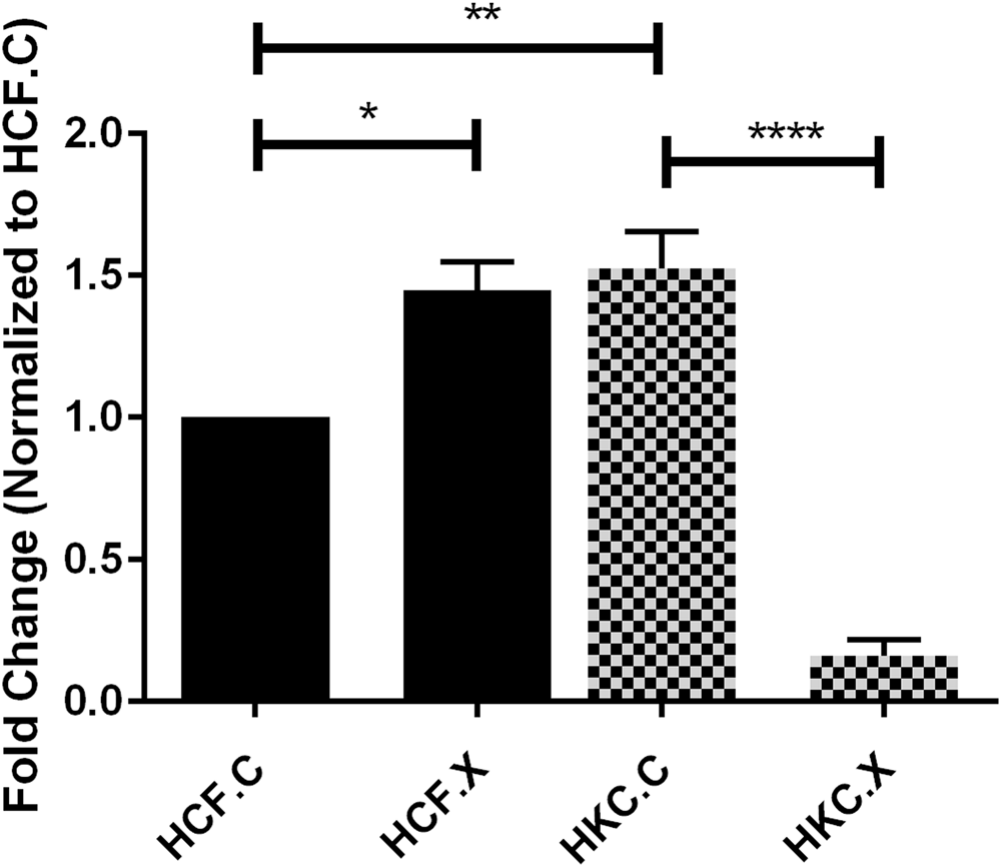
Protein expression for α-SMA in HCF, HKC controls, and HCF, HKCs treated with CXL Quantification of protein bands that are normalized to the loading control. n = 4, and error bars represent standard error of the mean. One way ANOVA was performed. *(P = 0.0466), **(P = 0.0030), ****(P = 0.0003).

The stability of the corneal stroma is critical for maintenance of corneal transparency. Severe KC can lead to the development of corneal scarring causing significant visual impairment^44^. Fibroblasts involved in scarring have a myofibroblast phenotype characterized by α-smooth muscle actin (α-SMA) expression. Therefore, we further investigated cellular differences by examining the expression of the fibrotic marker α-SMA pre/post CXL. Western blot analysis reveals a significant decrease in α-SMA expression in HKC.X 24 hours following CXL (*P = 0*.*0466*) compared to HKC.C. However, HCF.X revealed a significant increase in α-SMA expression when compared to HCF.C (*P = 0*.*0030*) (Fig. 6), (Supplemental Fig. 1).

#### Type I, Type III, and Type V collagens

**Figure 7:**
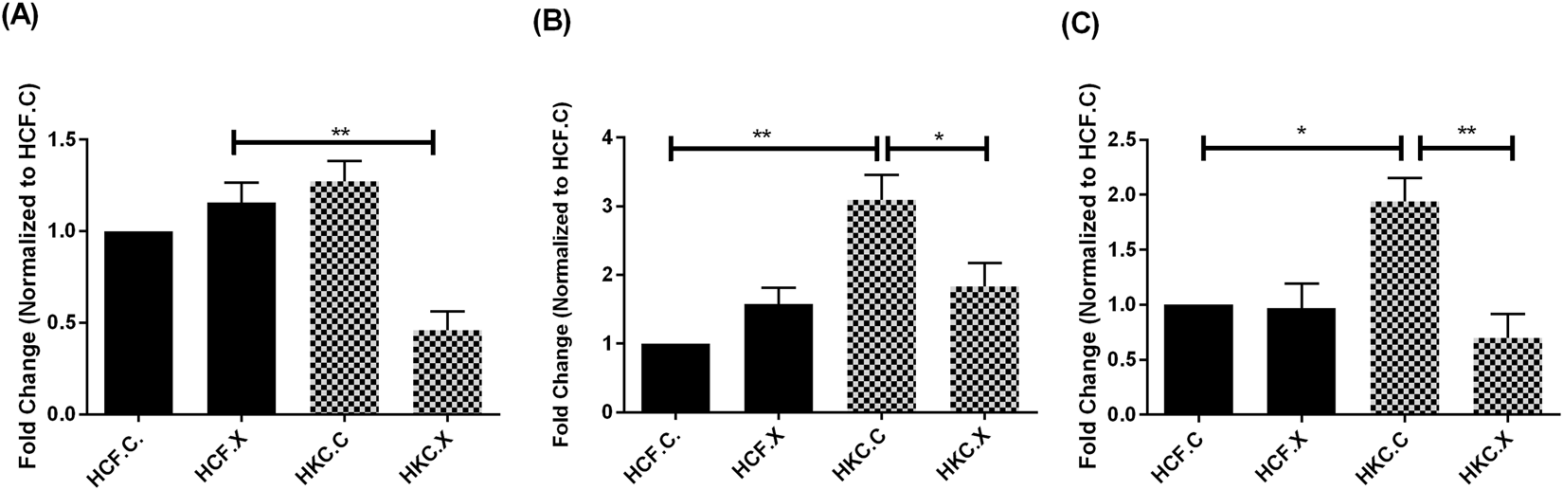
Collagen protein quantification post CXL: (**A**) Collagen I, **(P = 0.0078), (**B**) Collagen III, *(P = 0.0356), **(P = 0.0076), (**C**) Collagen V, *(P = 0.0142), **(P = 0.0097).

We further characterized the effect of CXL on ECM assembly by analyzing the expression of the key proteins, Collagen I, III, and V (Fig. 7), (Supplemental Fig. 2). HCF.X did not affect any of the collagen probes mentioned, while HKC.X (Fig. 7B) showed significant downregulation of Col III (*P = 0*.*0356*), as well as Col I (*P = 0*.*0078*), (Fig. 7A), and Col V (*P = 0*.*0097*), (Fig. 7C).

### Effect of CXL on regulatory SMAD6 and SMAD7 expression

**Figure 8:**
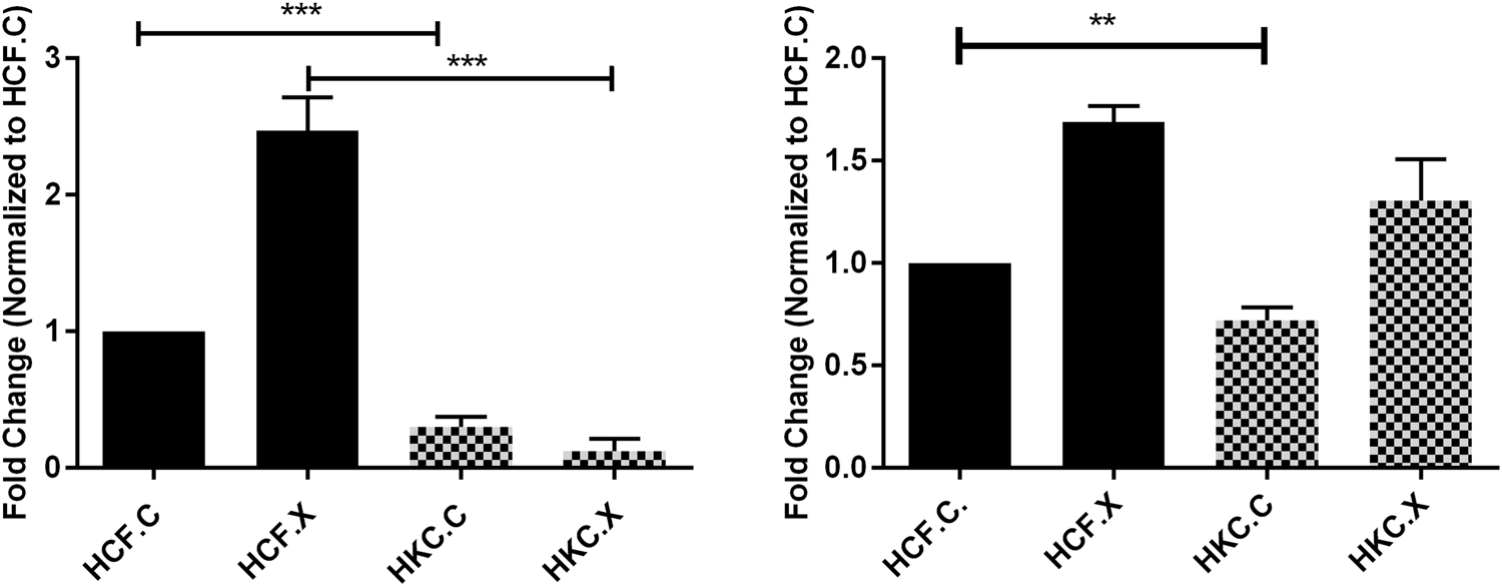
Quantification of SMAD6 and SMAD7 expression in HCFs and HKCs post CXL. Western blot analysis shows protein expression for (**A**) SMAD6, significant downregulation in HCF and HKC controls ***(P = 0.0003), similar pattern in HCF and HKC treated by CXL***(P = 0.0007). (**B**) SMAD7, significant downregulation shown in HKC control cells compared to HCF controls**(P = 0.0031).

We measured the gene expressions of the inhibitory SMADs (SMAD6 and SMAD7) to identify whether alterations in the basal levels following CXL treatment contribute to a more healthy ECM status, compared to the fibrotic phenotype observed in HKCs (Fig. 8), (Supplemental Fig. 3). Western blot analysis data showed HCF.X have a significantly upregulated expression of SMAD6 *(P = 0*.*0007)*, (Fig. 8A). However, SMAD7 expression was significantly downregulated in HKC.X (*P = 0*.*0031*) when compared to HCF.X (Fig. 8B). Our results show that HKCs express an altered level of the regulatory SMAD6 or SMAD7 expression levels with CXL treatment, suggesting a modulation effect of CXL on TGF-β downstream pathways^10^.

### SMAD2/SMAD3 altered expression post CXL

**Figure 9:**
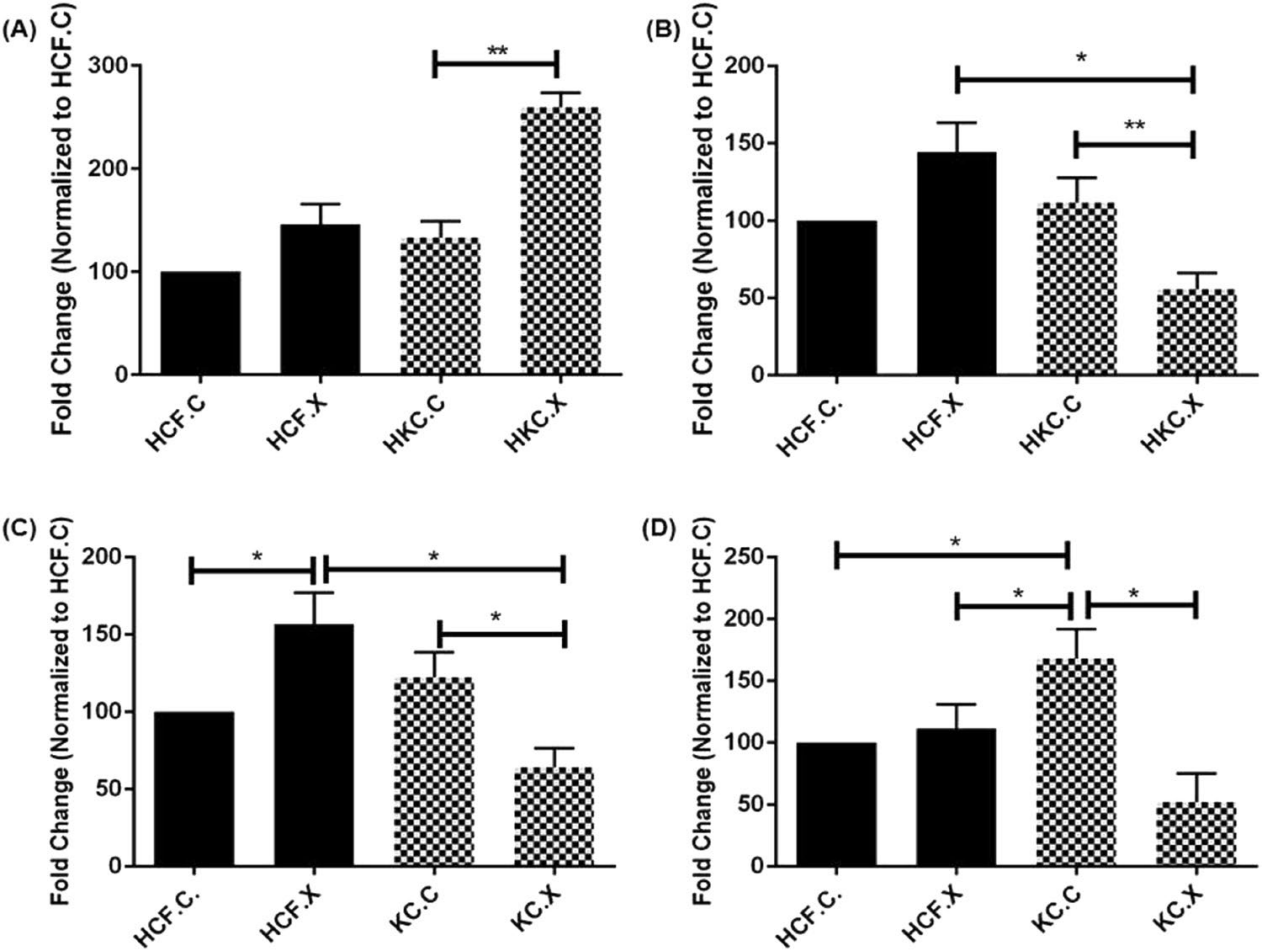
Quantification of protein expression for (**A**) SMAD2, (**B**) SMAD3, (**C**) pSMAD2, and (**D**) pSMAD3 following CXL treatment, n = 4. All samples were repeated at least three times. *p < 0.05 was considered to be statistically significant **p < 0.01.

To determine whether the altered expressions of regulatory SMAD6 and SMAD7 contribute further to TGF-β signaling modulation, we measured the protein expression level of SMAD2 and SMAD3 (Fig. 9), (Supplemental Fig. 4). SMAD2 was significantly upregulated in HKC.X (*P = 0*.*0195*), however, CXL treatment caused a significant downregulation in SMAD3 in HKC.X (*P = 0*.*0043*) compared to its respective control (Fig. 9A,B). HKC.X also showed a one-fold downregulation in both pSMAD2 and pSMAD3 expression *(P = 0*.*0077* and *P = 0*.*0081* respectively), compared to HKC.C (Fig. 9C,D). This data suggests a highly influenced downstream TGF-β signaling in response to CXL, leading to altered and more stable/healthy ECM assembly process.

### Lysyl Oxidase (LOX) Expression

**Figure 10:**
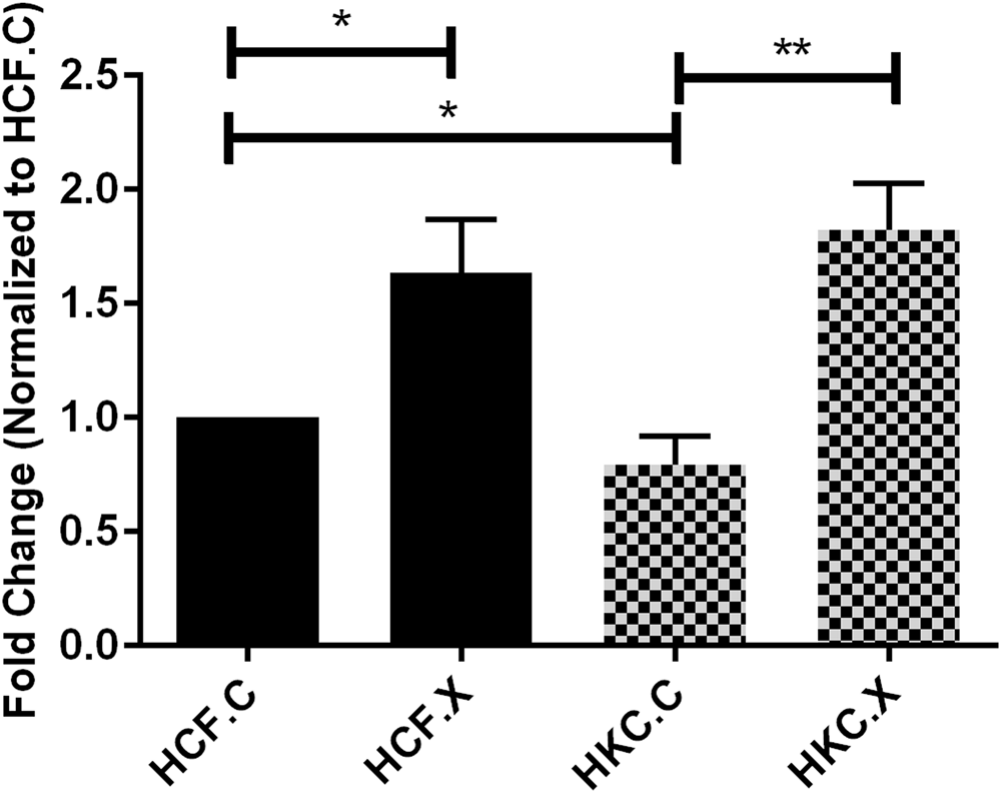
Protein expression for LOX in HCF, HKC controls, and HCF, HKCs treated with CXL. Quantification of protein bands are normalized to the loading control. N = 4, and error bars represent standard error of the mean. One way ANOVA was performed. *(P = 0.0120), **(P = 0.0015).

CXL for both collagen and elastin processes are mediated by LOX. In addition, hydroxylation can also play a key role in pathogenesis by determining which dysfunctional cross-links form preferentially^44^.We measured the expression of the LOX protein pre/post CXL (Fig. 10 and Supplemental Fig. 5). Several studies have previously shown that a decrease in LOX activity and changes in its modulation play a pivotal role in the defected collagen crosslinking in KC^17,45,46^. Not surprisingly, our data revealed a significant upregulation of this cross linking enzyme *(P = 0*.*0015)* in HKC.X.

## Discussion

KC is one of the main causes of cornea transplant. Current therapies based on the spectacles, contact lenses, and corneal transplantation^47^, simply correct the refractive errors of KC but do not treat the cause of the corneal ectasia and therefore cannot halt the progression of KC^48^. However, CXL is a therapeutical strategy based on targeting the underlying pathology of the disease, where long-wave UVA radiation aided by Riboflavin (vitamin B2)^22,31,49,50^, which acts as a photomediator, considerably increasing the absorption of UVA light on exposure to corneal stoma^22,23,51^. It has been demonstrated that absorption of UVA light within the lamellae of corneal stroma is approximately 30%, whereas combination with the photomediator properties of riboflavin increases this absorption from 30% to 95%^19^. Following exposure, riboflavin is excited into a triplet state thereby generating reactive oxygen species: singlet oxygen and superoxide anions then react with available groups nearby^32^. One of the most plausible mechanisms of CXL is thought to be the creation of additional chemical bonds between histidine, hydroxyproline, hydroxylysine, tyrosine, and threonine amino-acid residues^17,31^. Importantly, CXL can also cause cross-linking of other classes of macromolecules within the corneal stroma, such as proteoglycans, either to one another or to collagen molecules^28^.Collagen fibrils are linked together in a network pattern. LOX is the key enzyme for creating covalent bonds between these collagen fibrils^52^. Additionally, a linkage study of familial and case-control KC patients suggests that the LOX gene containing genomic loci may be associated with KC^17^, although pathogenic mutants were not found^46^. Unfortunately, it has yet to be determined how LOX activity is altered in KC and whether the deregulation contributes to the pathogenesis of this progressive disorder. Our data shows an important correlation of LOX activity and CXL with a decrease in collagens I, -III, and -V in HKCs. However, the precise mechanism of CXL at the molecular level has not yet been elucidated and the treatments are not tailored to each patient.

In this study, we focused on investigating the microstructural changes occurring in HKCs after CXL. Wollensak *et al*.^24^ reported an abrupt cytotoxic effect on porcine keratocytes *in vitro* using CXL, and a decrease in viability and an increase of apoptosis of porcine keratocytes^53^. Previous studies^31,54^ have suggested that the advanced compact corneal structure due to KC cell apoptosis, increased interfibrillar and intrafibrillar chemical bonds, and ECM remodeling may explain a thinner cornea post CXL. At present, there is paucity of information regarding corneal cell apoptosis at the cellular-subcellular level and alterations of the apoptotic pathways in normal and KC cells have to be described pre/post CXL^51,55^, so that we are better able to understand the disease pathology.

The impact of riboflavin-UVA induced CXL has also been reported in a human *ex vivo* keratoconus corneas^22,51^. However, ex vivo and 2-D *in vitro* system differ from primary human corneal cells grown in a 3-D *in vitro* model. 2-D cultures are optimized to sustain cell growth and organization on the surface of the material^56^. In comparison, 3-D culture systems provide closer resemblance of native tissue architecture, and further guide cell organization and tissue development. Therefore, the dimensionality of the culture environment strongly affects cellular organization and responses. Our present study showed a significant increase in the myofibroblast marker, α-SMA expression, which plays a critical role in corneal wound healing and fibrosis^3,47,57^. α-SMA and the forces generated by the contractile activity of myofibroblasts are transmitted to the surrounding ECM through specialized focal adhesions containing transmembrane integrins^58^, leading to a more compacted ECM^59^. Considering the clinical impact CXL has by stiffening the corneal tissue^16,24,60^, thereby stabilizing KC progression, it is critical to fully understand the cellular and molecular mechanisms so that we can further improve in CXL techniques.

Experimental studies have shown numerous Ki-67-positive fibroblasts shortly after CXL^61^, whereas only a few α-SMA-positive myofibroblasts were detected in the central CXL region^62^.This evidence indicates that the activation of keratocytes after corneal CXL occurs mainly by means of their transformation into fibroblasts^61,63^. This may explain why mild or no opacities have been observed after CXL in the above-mentioned studies^31,64^, considering that the degree of opacity correlates directly with the number of activated keratocytes^32,53^. Since the composition of the stromal ECM is tightly regulated and ultimately defines the structural integrity of the cornea^3,65^, we investigated the effect of CXL on collagen composition in the corneal stroma. Our data showed that CXL significantly decreased collagen III in HKCs and revealed a lower expression level of both collagen I, and V. Thus, maintaining a healthy ECM phenotype^66^. This positive impact of CXL on the corneal stroma is critical in order to sustain the mechanical strength needed to form the anterior segment of the eye, whilst maintaining the high degree of transparency required for light transmission^19^.

Furthermore, we determined the cell migratory properties of these cells which are one of the crucial factors that determines effective wound healing and normal function of the cornea. Among the many growth factors studied in the context of wound healing, transforming growth factor beta (TGF-β) is thought to have the broadest spectrum of effects^67^. Many of the molecular mechanisms underlying the TGF-β/Smad signaling pathway have been well characterized^7,10,68^. Therefore, studying the effect of the therapeutic CXL strategy on the various inhibitory, as well as regulatory SMADs, exposes the possibility of targeting TGF-β signaling pathway to improve wound healing and/or reduce scarring in KC patients^37^. Thus, with the aid of our novel 3-D *in vitro* CXL model, this study suggests that CXL treatment leads to significant downregulation of the vast majority of the corneal collagens (-I, -III, -V), which could explain why minimal opacities have been observed in clinical studies following CXL.

Future studies, could include the corneal epithelial layer (epi-on) in order to compare the two techniques (epi-on versus epi-off). Long term CXL effects and repeated CXL applications could also be investigated using our 3D model. Conclusively, our model, will aid future refinement in CXL techniques and allow for more targeted clinical procedures.

## Materials and Methods

### Ethics

All procedures used in these studies adhered to the tenets of the Declaration of Helsinki. All methods were carried out in accordance with the relevant guidelines and regulations. Healthy human corneas were obtained from the National Disease Research Interchange (NDRI, Philadelphia, PA). Keratoconus donor corneas were obtained from our clinical collaborators Drs. Hjortdal (Aarhus University Hospital, Aarhus, Denmark), and Garett (Dean McGee Eye Institute). Institutional review board (IRB) approval was received prior to initiation of experiments described in this study both at Aarhus University Hospital and the University of Oklahoma Health Sciences Center- Dean McGee Eye Institute (IRB protocols #1-10-72-77-14 and # 3450, respectively) with written informed consent obtained from patients. Inclusion/exclusion criteria for data collection were established at the onset of data analysis. Inclusion criteria for healthy controls required absence of KC diagnosis or other corneal diseases. Inclusion criteria for KC patients required diagnosis of KC by a certified ophthalmologist and absence of other ophthalmic conditions, and to exclude clinical data from patients who had previously received collagen crosslinking or undergone penetrating keratoplasty.

For this study, HCFs were isolated from three different donors with average age 58 ± 13.6 y/o and three KC different donors with average age 58 ± 12.9 y/o. Each one of them was tested at least three times using the model described here.

### Cell Isolation

Healthy and KC human corneas were processed as previously described^4,69^. Through brief scraping with a razor blade, the endothelium and epithelium were removed from the stroma. The stromal tissue was cut into small pieces (4 to 5 pieces of 2 mm × 2 mm). The pieces of stroma were allowed to adhere to the bottom of a T25 flask for 30 minutes at 37 °C before carefully adding Eagle’s Minimum Essential Media (EMEM: ATCC: Manassas, VA) containing 10% Fetal Bovine Serum (FBS: Atlantic Biologic’s; Lawrenceville, CA) and 1% Antibiotic (Gibco® Antibiotic-Antimycotic, Life technologies) to the flask without disturbing the ex-plants. At approximatelly100% confluency, explants were further passaged into T75 flask and incubated at 37 °C, 5% CO_2_ for further expansion.

### 3-D Cell Culture and ECM Assembly

HCFs and HKCs were seeded on a transwell 6-well plates with polycarbonate membrane inserts with 0.4-μm pores (Transwell; Corning Costar; Charlotte, NC) at a density of 1 × 10^6^ cells/well and cultured in a 10% FBS EMEM medium and 1% Antibiotic, stimulated with 0.5 mM 2-O-α-D-Glucopyranosyl-L-Ascorbic Acid (Vitamin C, American Custom Chemicals Corporation, San Diego, CA). Cultures were grown for 4 weeks before further processing, and fresh media was supplied every other day for the duration of the experiment^4,7,69^.

### Corneal Collagen Cross-Linking

We further developed our 3-D *in vitro* model to accommodate Riboflavin-UVA-CXL, establishing a setup mimicking the current clinical treatment of KC. In this model, both cell types (HCFs and HKCs) were plated on transwell polycarbonate membrane inserts, and at week 4 time point^4^ a mixed riboflavin 0.1% PBS solution was added to the constructs followed by UVA irradiation to ensure riboflavin cell saturation. Using a UV-X illumination system (version 1000; IROC AG, Zurich, Switzerland) shown in (Fig. 1) at a wavelength of 360–370 nm and an irradiance of 3 mW/cm^2^ of UVA^20^, with a total energy dose of 5.4 J/cm^2^, which was calibrated prior to each treatment using a UVA meter (LaserMate-Q; LASER 2000, Wessling, Germany). Each well was exposed to UVA for 3 minutes at a 3 cm distance, mirroring current CXL clinical settings^24^. Post irradiation, each construct was rinsed with PBS and incubated in fresh media for 12 h before further analysis.

### Western Blot

Western blot (WB) analyses of both HCFs and HKCs were performed with lysis of cells, as previously described^9,10^. Protein concentration and purity were assessed by Bradford assay (Thermo Scientific, IL). 4–20% Tris-Glycine gels (Novex, Life technologies, Carlsbad, CA) was used for gel electrophoresis, to which equal amounts of proteins were loaded and a protein transfer was done using Nitrocellulose membrane (Novex, Nitrocellulose membrane filter par sandwich, Life Technologies). After incubation in a 5% BSA blocking solution (Thermo Scientific, IL), the membranes were incubated with primary rabbit antibodies: Collagen I (ab34710; Abcam, Cambridge, MA, USA), Collagen III (ab7778; Abcam, Cambridge, MA), Collagen V (ab94673; Abcam, Cambridge, MA), anti-SMAD 2, 3 (Invitrogen, Camarillo, CA), anti-SMAD7 (Sigma-Aldrich, Saint Louis, MO), anti-SMAD4 (Abcam, Cambridge, MA), anti-SMAD6 (Abcam, Cambridge, MA), anti-α SMA (Abcam, Cambridge, MA). After primary incubation, the membrane was washed for 5 min (3×) in Tris-buffered Solution with Tween20 before probing with secondary antibody Goat anti-Rb Alexafluor 568 (Life Technologies, Grand Island, NY, USA) at 1:2000 dilution for 1 h with rocking at room temperature. The membrane was allowed to dry before imaging using ChemiDoc-it to image. GAPDH (Abcam, Cambridge, MA) was used as the loading control and results were analyzed by normalizing the value to that of the loading control expression and plotting the fold expression.

### Cell Viability (Photo toxicity)

To determine the effects of CXL on cell viability, a live/dead® Viability Assay kit (LIVE/DEAD Kit for mammalian cells, Molecular Probes, Eugene, OR) was used. As previously describe^70^, this assay is based on the simultaneous determination of live and dead cells with two probes that measure known cell viability parameters; plasma membrane integrity and intracellular esterase activity. 3-D constructs with HCFs (controls (HCF.C), CXL treated (HCF.X)) and HKCs (controls (HKC.C), CXL treated (HKC.X)) seeded at a density of 1 × 10^6^ cells/well onto 3-D *in vitro* polycarbonate membranes were stained with 4 μmol/L calcein AM and 2 μmol/L ethidium homodimer-1 in phosphate-buffered saline for 30 minutes at room temperature in the dark. Cells were analyzed by fluorescence microplate reader (CLARIOstar monochromatic microplate reader-BMG Labtech) which provided excitation at 494/517 nm and emission at 528/617 nm.

### Cell Proliferation

Cellular proliferation pre and post CXL was determined using vibrant MTT cell proliferation kit (Life Technologies, USA, Cat # 13154) 24 h after CXL. HCFs and HKCs were seeded in a 96-multiwell plate at a density of 10000 cells/well in 100 *μ*L of culture medium. Following 24 hours of culture 10 μl of the 12 mM MTT stock solution was added to each well and incubated for 4 hours at 37 °C as per the manufacturer’s protocol. Thereafter, 100 μl of the SDS-HCl solution was added to each well and evenly mixed, followed by an incubation period of 4 hours at 37 °C in a humidified chamber. Samples were mixed again and the absorbance was measured at 570 nM using the plate reader. The results were analyzed and processed in Graph Pad Prism 6 (GraphPad Software, CA) to determine the cellular proliferation rate.

### Desiccator and Corneal hydration percentage

In order to evaluate the immediate effect of CXL on corneal hydration and stiffness *in vitro*, all constructs (HCFs and HKCs) underwent initial weighting at the end of week 4, followed by dehydration for 48 h in a desiccator and weighed again to obtain their dry mass^42^. Hydration (H%) of each construct was calculated as follows: H% = (wetmass-drymass)/wet mass*100^16^. Samples from both cell types were normalized to the HCF controls.

### Assessment of Cell Migration

Cell migration was assessed using an *in vitro* scratch assay as previously described^43^. Briefly, 1 × 10^6^ cells/well for both cell types (HCFs, and HKCs) were seeded in six well plates and allowed to achieve 100% confluency. Scratches were performed pre/post CXL technique using a sterile 200 micropipette tip through the cell monolayer and rinsed with PBS. Fresh medium was added and plates were imaged at pre-determined time points (0 hr, 4 hr, 12 hr, 24 hr and 48 hr). The migration pattern was evaluated and quantified using ImageJ software.

### Statistical Analysis

Statistical analysis was performed using GraphPad Prism 6 and a one-way ANOVA and Mann-Whitney unpaired T-test, where applicable. P values below 0.05 were considered statistically significant.

### Data availability

All data is included in this manuscript and is freely available.

## Electronic supplementary material

Supplementary Info
