# Supplementary material for: Human in vitro Model Reveals the Effects of Collagen Cross-linking on Keratoconus Pathogenesis
Source: Sci Rep. 2017 Oct 2;7:12517. doi: 10.1038/s41598-017-12598-8 (PMC5624875; doi:10.1038/s41598-017-12598-8)
Supplement: Supplementary file 1 — Supplementary Info [file 41598_2017_12598_MOESM1_ESM.doc]

**Human *in vitro* Model Reveals the Effects of Collagen Cross-linking on Keratoconus Pathogenesis**

Rabab Sharif^1^, Jesper Hjortdal^2^, Henrik Sejersen^2^, Garett Frank^3^, Dimitrios Karamichos^1,3*^

^1^Department of Cell Biology, University of Oklahoma Health science Center, Oklahoma City, Oklahoma, USA ^2^Department of Ophthalmology, Aarhus University Hospital, Aarhus C, Denmark ^3^Department of Ophthalmology/Dean McGee Eye Institute, University of Oklahoma Health Science Center, Oklahoma City, Oklahoma, USA

* Correspondence to: Dimitrios Karamichos, Department of Ophthalmology/Dean McGee Eye Institute, University of Oklahoma Health Sciences Center, Oklahoma City, OK 73104; Phone: (405) 271 4019; FAX: (405) 271 8128; email: [Dimitrios-Karamichos@ouhsc.edu](mailto:Dimitrios-Karamichos@ouhsc.edu).

**
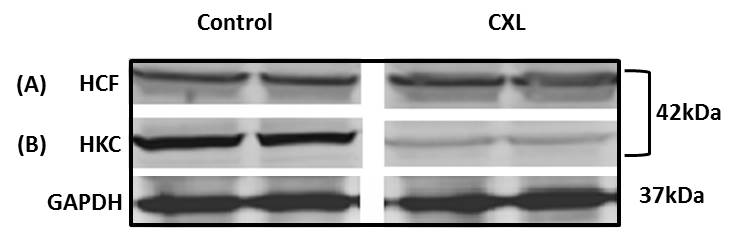
**

**Supplemental Figure 1.** Representative western blot images documenting the decreased expression of α-SMA in HKCs (B1.CXL) following Collagen crosslinking compared to HCFs controls and HCFs CXL (A1), as well as HKCs controls (B1.Control)


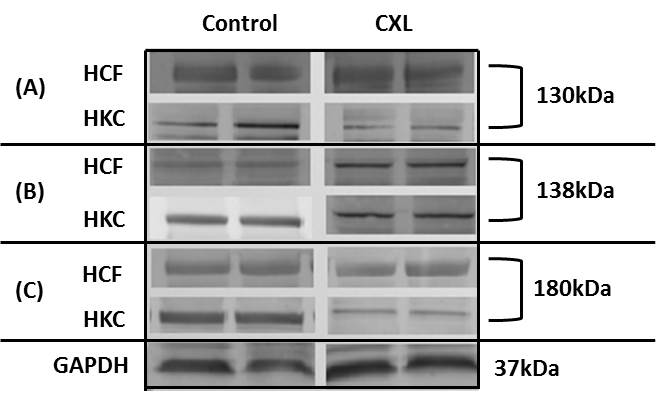


**Supplemental Figure 2**. Representative western blot images and analysis of: collagen I (130kDa) (A2), collagen III (138kDa) (B2), and collagen V (180kDa) (C2) expression levels, and housekeeping gene GAPDH (37kDa) following collagen crosslinking in HCFs and HKCs.


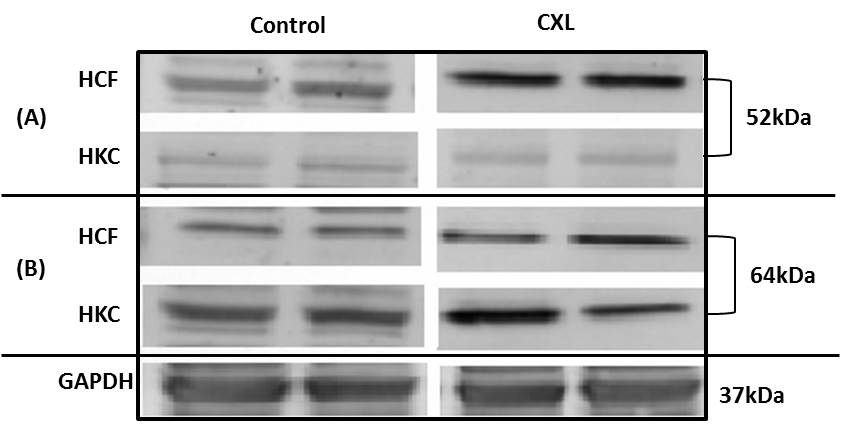


**Supplemental Figure 3.** Representative western blots show protein expression for SMAD6 (52kDa) (A3), and SMAD7 (64kDa) (B3), and Housekeeping gene GAPDH (37kDa), following CXL.

**
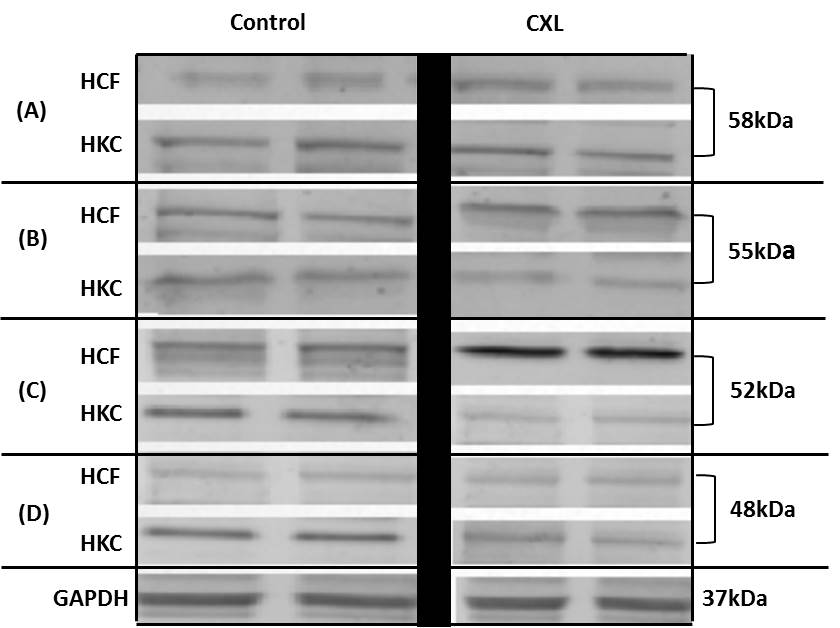
**

**Supplemental Figure 4**. Representative western blots show protein expression of SMAD2 (58kDa) (A4), SMAD3 (55kDa) (B4), pSMAD2 (52kDa) (C4), and pSMAD3 (48kDa) (D4), and Housekeeping gene GAPDH (37kDa), in HCF, HKC controls, and HCF, HKCs treated with CXL.


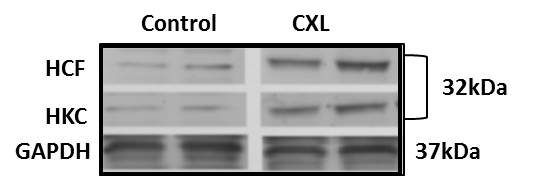


**Supplemental Figure 5.** Representative western blots show protein expression for LOX (32kDa), and Housekeeping gene GAPDH (37kDa) in HCF, HKC controls, and HCF, HKCs treated with CXL.
